# Supplementary material for: The ESCRT-III isoforms CHMP2A and CHMP2B display different effects on membranes upon polymerization
Source: BMC Biol. 2021 Apr 8;19:66. doi: 10.1186/s12915-021-00983-9 (PMC8033747; doi:10.1186/s12915-021-00983-9)
Supplement: Supplementary file 1 — Additional file 1: Figure S1. Evolution of the ESCRT-III complex. (A) Table illustrating the ESCRT-III complex function, origin and homologs in S. cerevisiae and H. sapiens. (B) Distribution of Vps2 and Vps24 genes across Eukaryotes showing the presence of two Vps2 genes in high organisms. (C) Table illustrating the implication of ESCRT-III subunits in different subcellular locations in S. cerevisiae and H. sapiens. The names are the human homologs in case of S. cerevisiae. [file 12915_2021_983_MOESM1_ESM.pdf]

SUPPLEMENTARY 1

A

ESCRT complex	Proposed Function	Evolutionary origin	Yeast	Human
**ESCRT -III**	Membrane remodelling	Archaea	Vps20<br>Snf7<br>Vps24<br>Vps2	CHMP6<br>CHMP4A, B, C<br>CHMP3<br>CHMP2A, B

B

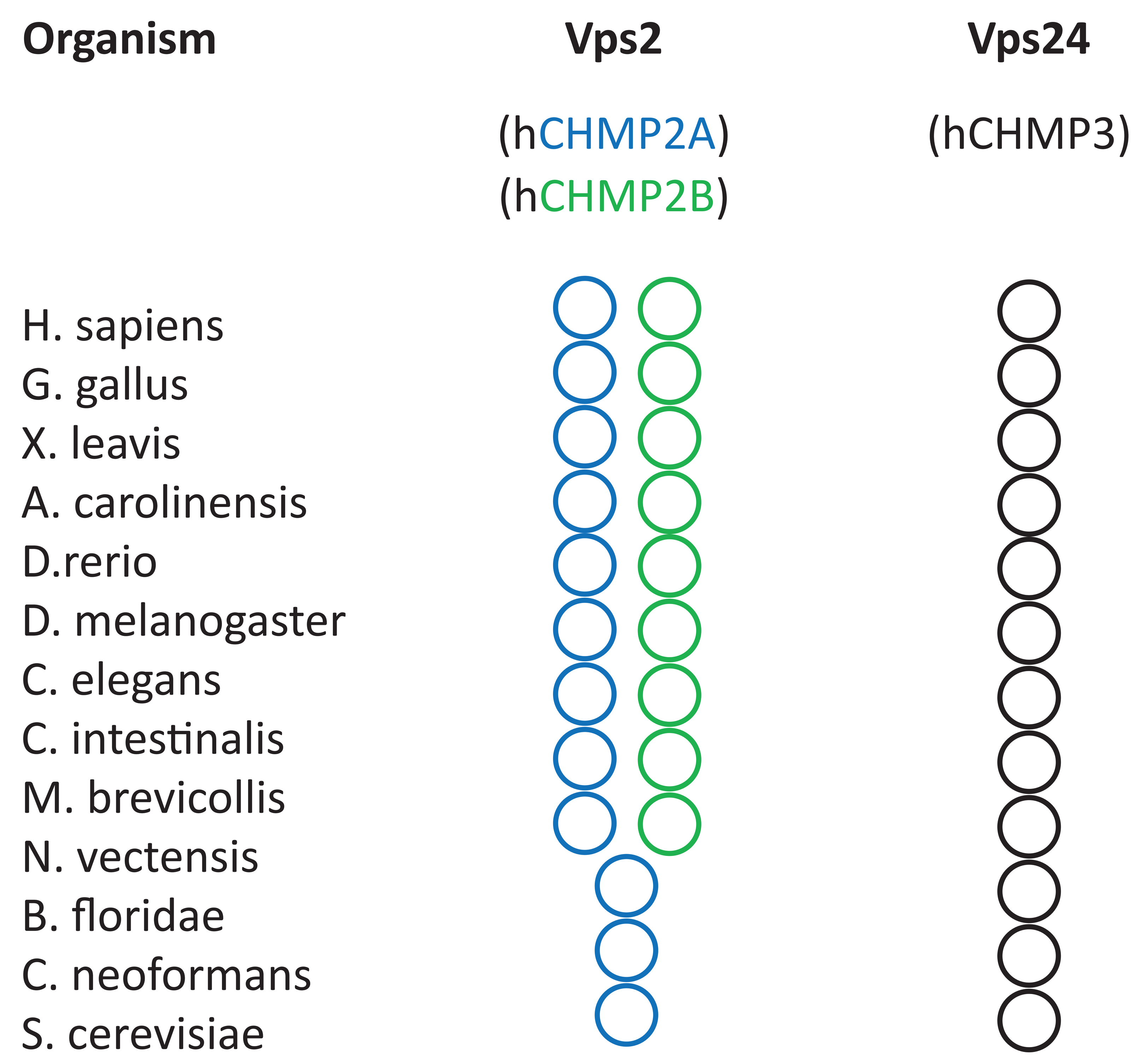

C

Cellular process	Organism	ESCRT -III core components
**MVB formation**	S. cerevisiae<br>H. sapiens	CHMP4B - CHMP3<br>CHMP2A
**Cytokinesis**	H. sapiens	CHMP4B - CHMP3<br>CHMP2A - CHMP2B
**HIV-1 budding**	H. sapiens	CHMP4B - CHMP3<br>CHMP2A - CHMP2B
**Neurone severing**	H. sapiens	CHMP4B - CHMP3<br>CHMP2A - CHMP2B
**Pasma membrane repair**	H. sapiens	CHMP4B - CHMP3<br>CHMP2A - CHMP2B
