# Supplementary figures and images for: The ESCRT-III isoforms CHMP2A and CHMP2B display different effects on membranes upon polymerization
Source: BMC Biol. 2021 Apr 8;19:66. doi: 10.1186/s12915-021-00983-9 (PMC8033747; doi:10.1186/s12915-021-00983-9)

SUPPLEMENTARY 2

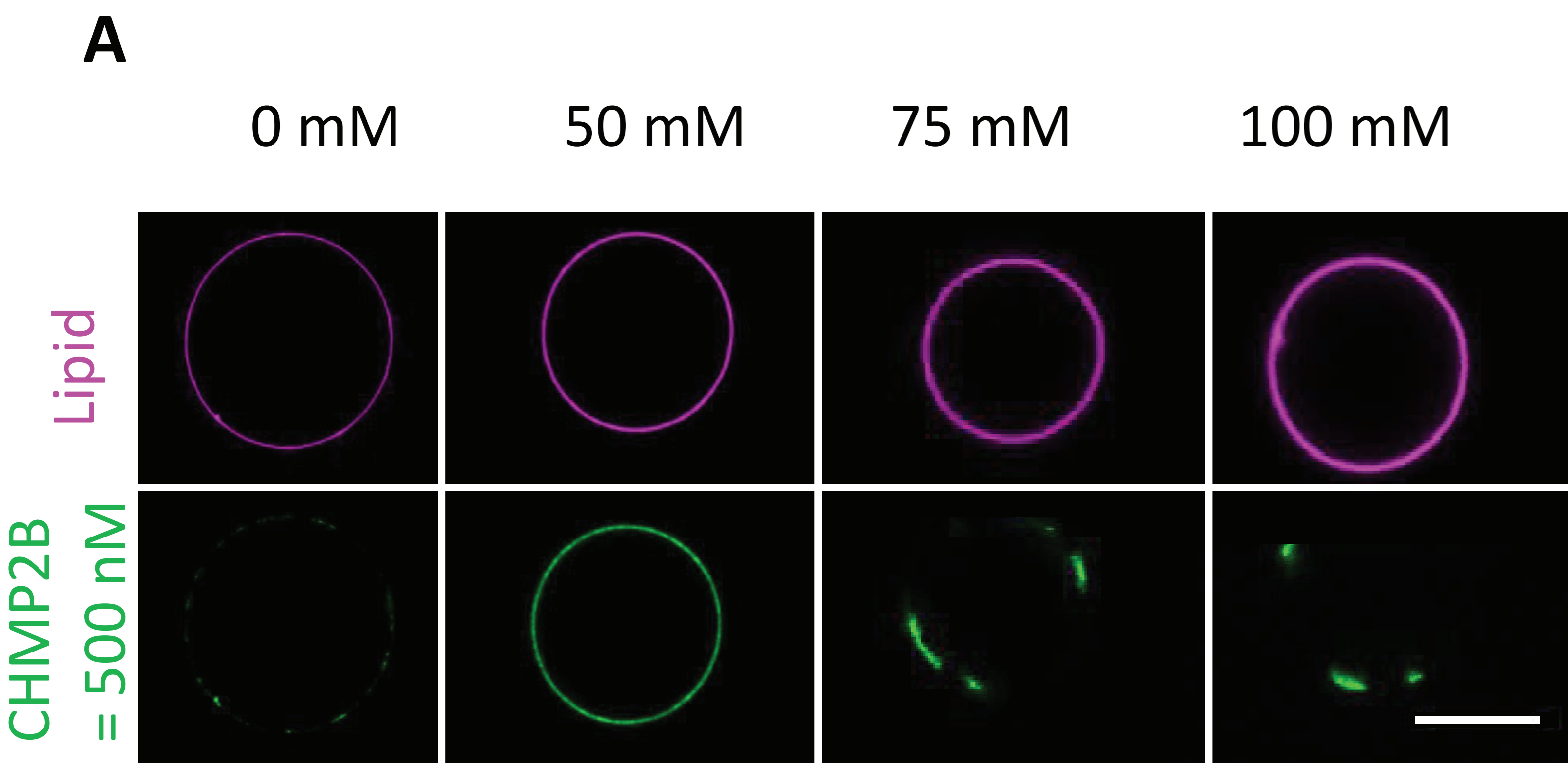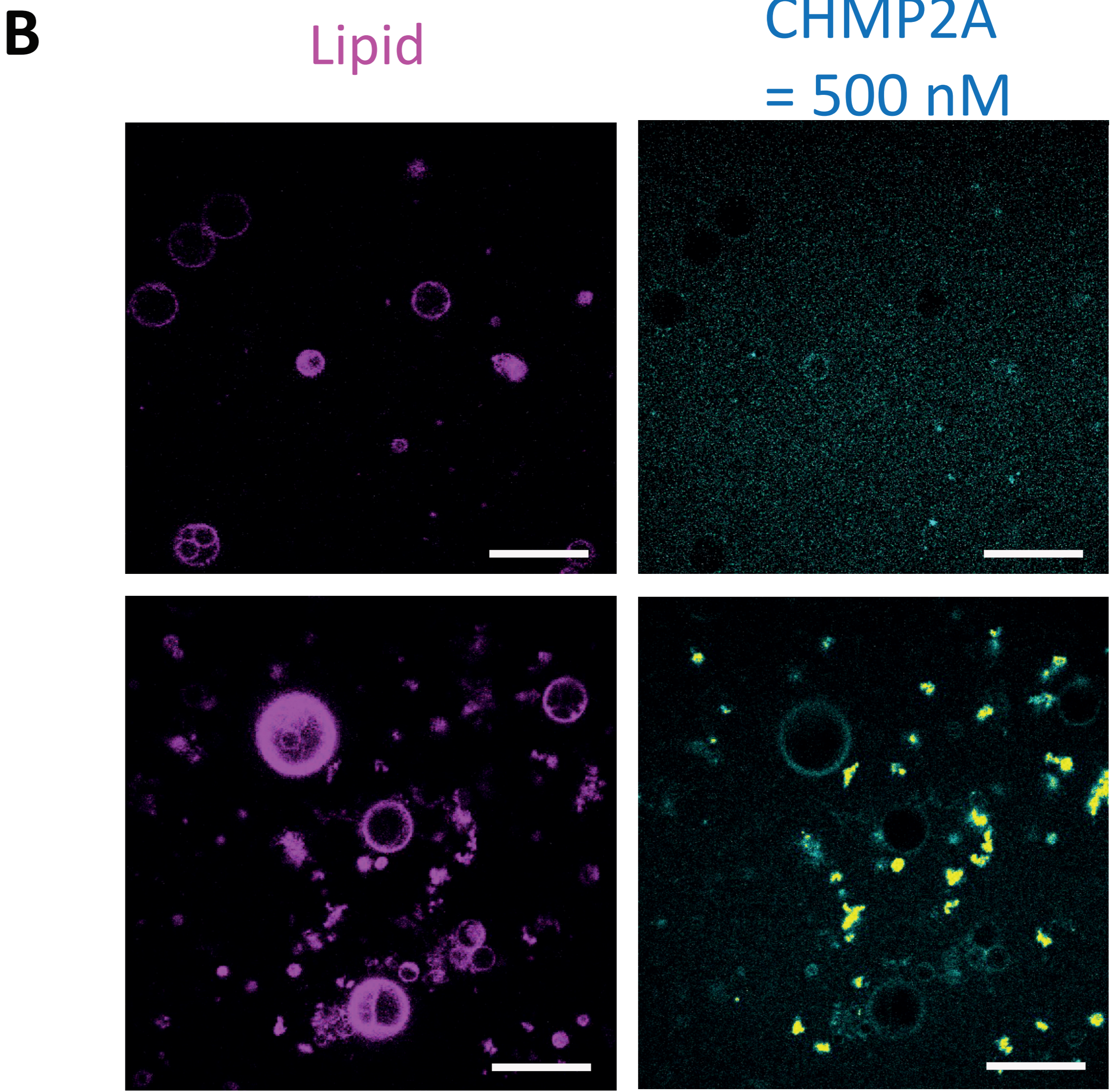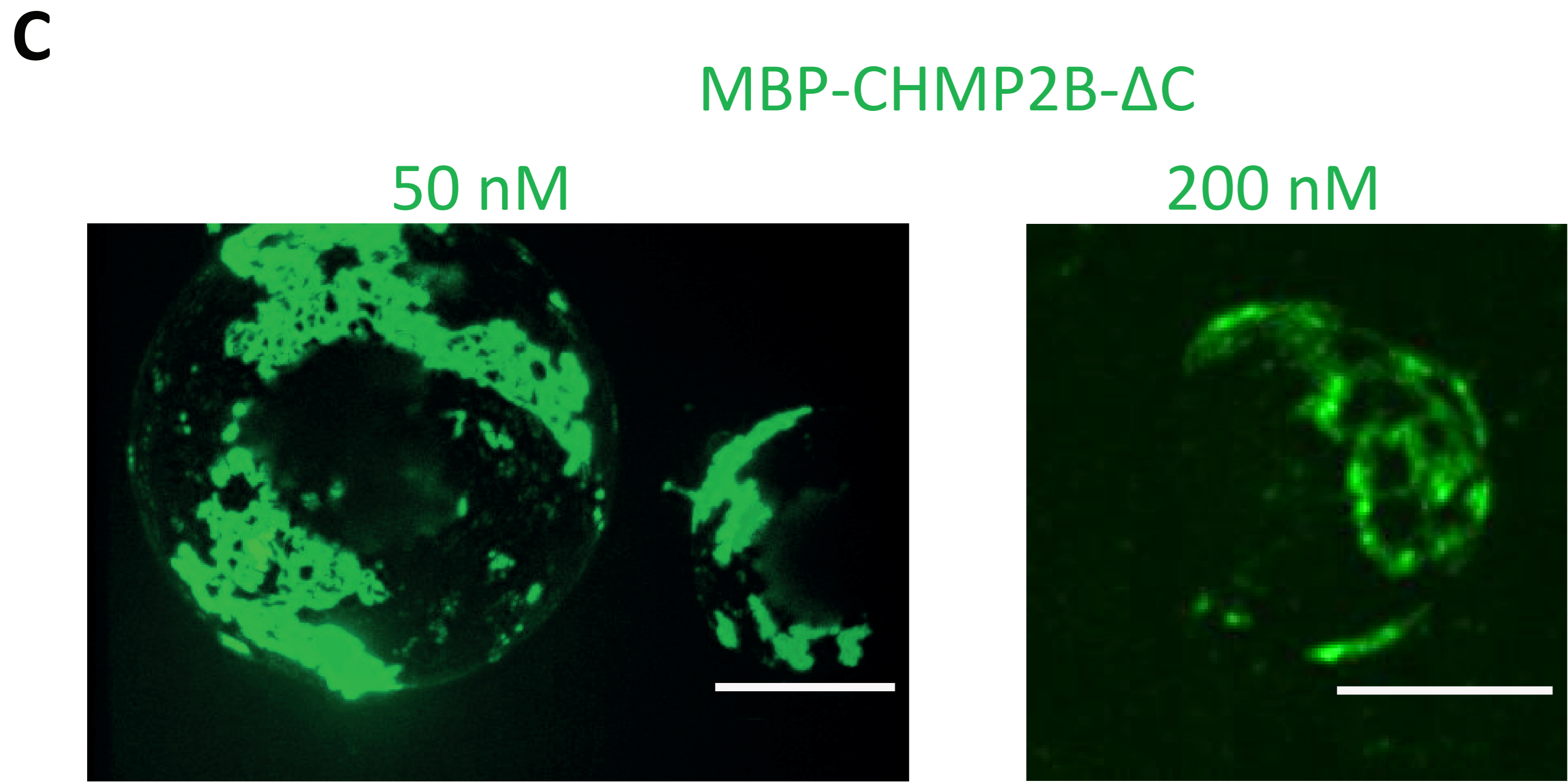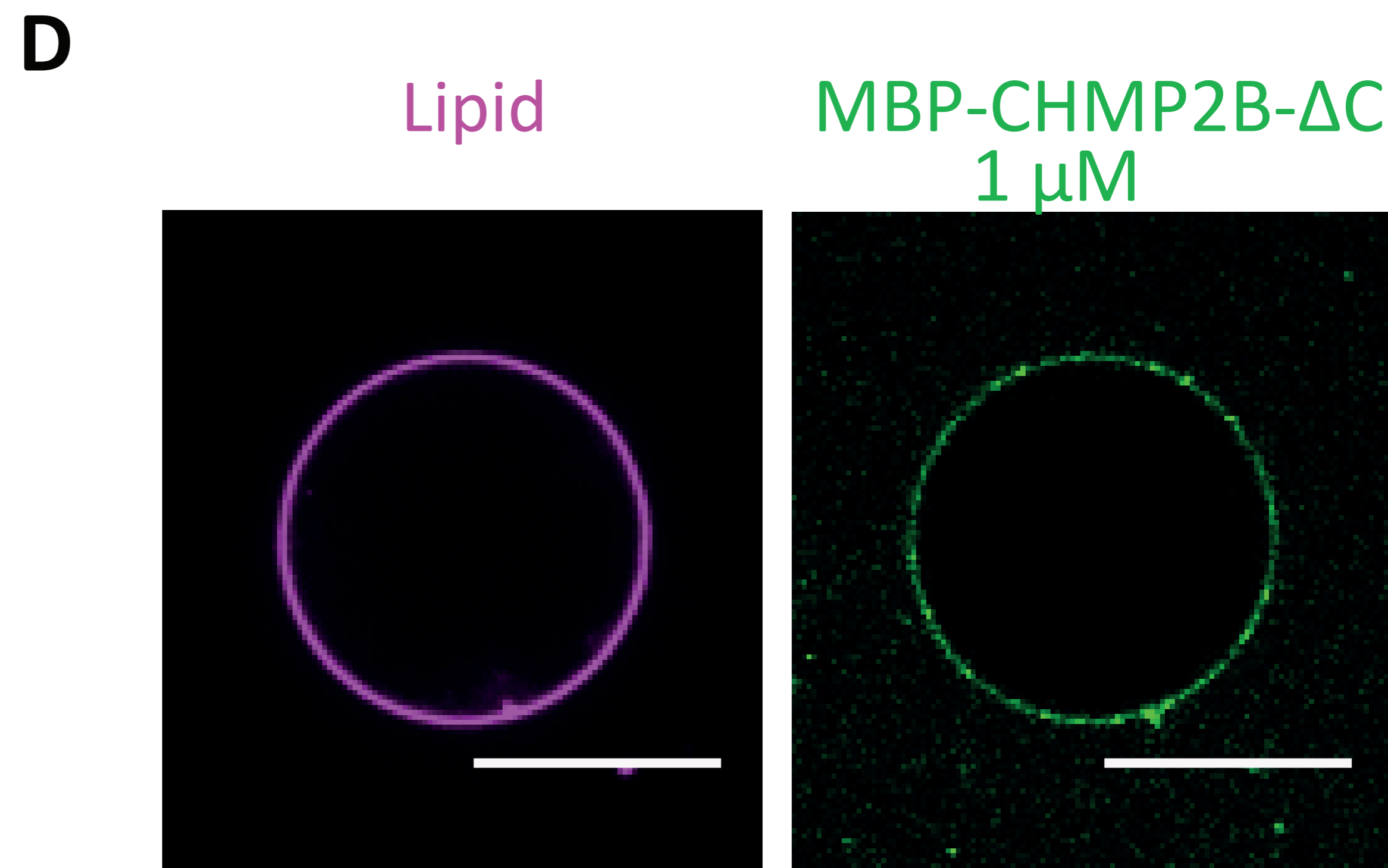

Supplement: Supplementary file 2 — Additional file 2: Figure S2. Study of CHMP protein-membrane interaction. (A) Optimization of the buffer conditions to optimize the binding of CHMP2B-ΔC (noted here CHMP2B) at 500 nM. Pre-formed vesicles were incubated with CHMP2B-ΔC in buffers with different salt concentrations ranging from 0 mM to 100 mM NaCl (+Tris 25 mM at pH 7.5) and imaged with confocal microscope after 30 min incubation. Lipid signal is shown in magenta and protein signal in green. Scale bar: 5 μm. (B) Confocal image of MBP-CHMP2A-ΔC (noted here CHMP2A) without TEV (Top line) and in the presence of TEV to cleave the MBP tag (Bottom line). Saturated protein fluorescent signal is represented in yellow. Cleavage of MBP tag slightly increases the interaction but induces aggregation. Scale bar: 30 μm. (C) Spinning disk image of GUV incubated with MBP-CHMP2B-ΔC (noted here MBP-CHMP2B) at a concentration of 50 nM (left image, scale bar = 10 μm) and at 200 nm (right image; scale bar = 5 μm). (D) Confocal images of GUV incubated with MBP-CHMP2B-ΔC at a concentration of 1 μM (noted here MBP-CHMP2B), submitted to an osmotic pressure difference equal to 150% (Osmolarity inside and outside the GUV are respectively 120 mOsm.L− 1 and 315 mOsm.L− 1). Scale bar: 10 μm. [file 12915_2021_983_MOESM2_ESM.pdf]

SUPPLEMENTARY 3

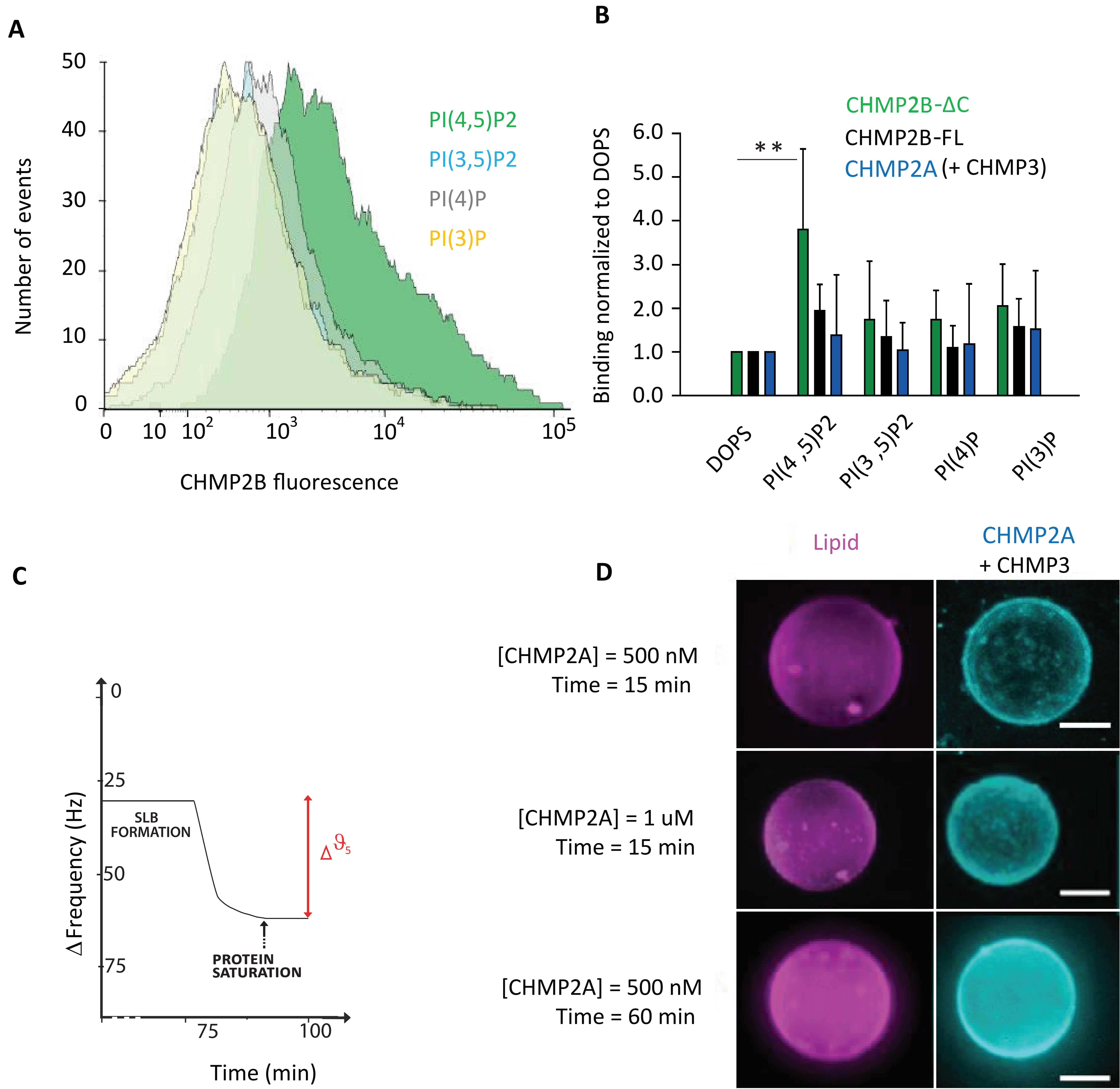

Supplement: Supplementary file 3 — Additional file 3: Figure S3. (A) Histograms of CHMP2B-ΔC protein fluorescence intensity for PI(3)P, PI(4)P, PI(3,5)P2 and PI(4,5)P2 GUVs (lipid composition 2). (B) Comparison of the binding density of MBP-CHMP2A-ΔC + CHMP3 and of CHMP2B-ΔC to GUVs with different charged lipids, measured by FACS, corresponding to Fig. 1d. The values are normalized to their respective binding density to DOPS. ** = p-value< 0.01 (Student’s t-test). N = 4 (number of FACS experiment with about 104 counted events per experiment, per condition). (C) QCM-D experiment displaying the typical frequency shift of − 25 Hz after supported bilayer formation and a frequency shift Δυ5 representative of the amount of protein bound to the bilayer. (D) Spinning disk images of interaction of MBP-CHMP2A-ΔC + CHMP3 in BP buffer on 10% PI(4,5)P2-containing GUVs. CHMP2A-ΔC fluorescent signal is displayed. A z-projection is represented. The different panels corresponding to 3 representative GUVs show the homogeneous coverage of the co-polymer as a function of protein concentration and incubation time. First panel: CHMP2A and CHMP3 are incubated at 500 nM and 2 μM, respectively, for 15 min. Second panel: CHMP2A and CHMP3 are incubated at 1 μM and 4 μM, respectively, for 15 min. Third panel: CHMP2A and CHMP3 are incubated at 500 nM and 2 μM, respectively, for 60 min. Scale bar, 10 μm. [file 12915_2021_983_MOESM3_ESM.pdf]

SUPPLEMENTARY 5

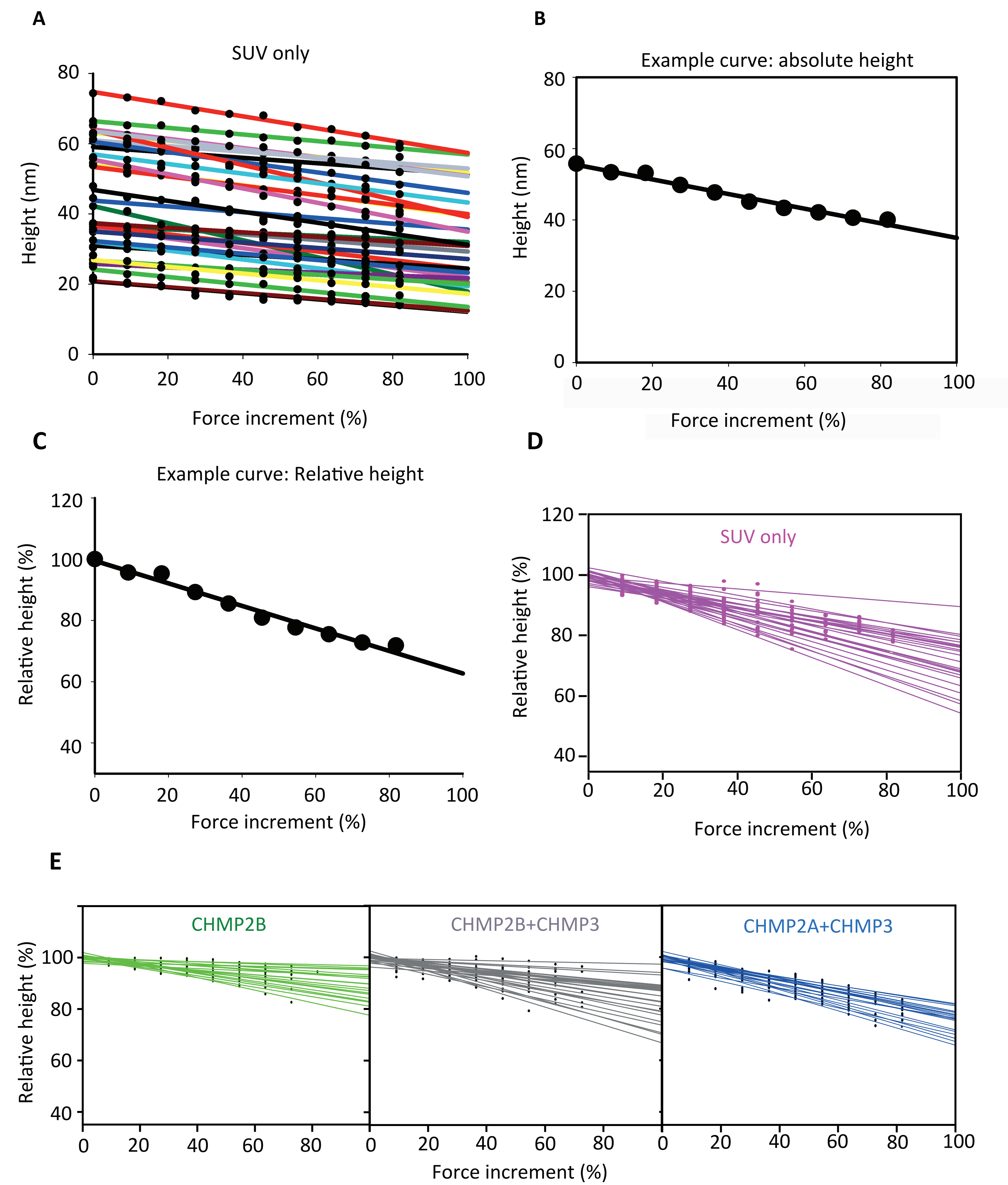

Supplement: Supplementary file 6 — Additional file 6: Figure S5. Deformation of bare vesicles and vesicles covered with CHMP proteins. (A) Reduction of vesicle height under increasing force for bare vesicles. ‘Zero’ force increment represents the minimum imaging force (~ 150 pN). (B) Example of deformation for a ~ 60 nm vesicle over increasing force up to 80% of the initial imaging force. (C) Represents the transformation of vesicle height to relative height for each point for the curve in D. (D) represents the relative height vs force increment for all the curves from panel A for bare SUVs. (E) represents the relative height vs force increment for SUV covered with CHMP2B (left), CHMP2B + CHMP3 (middle) and CHMP2A + CHMP3 (right). [file 12915_2021_983_MOESM6_ESM.pdf]

SUPPLEMENTARY 6

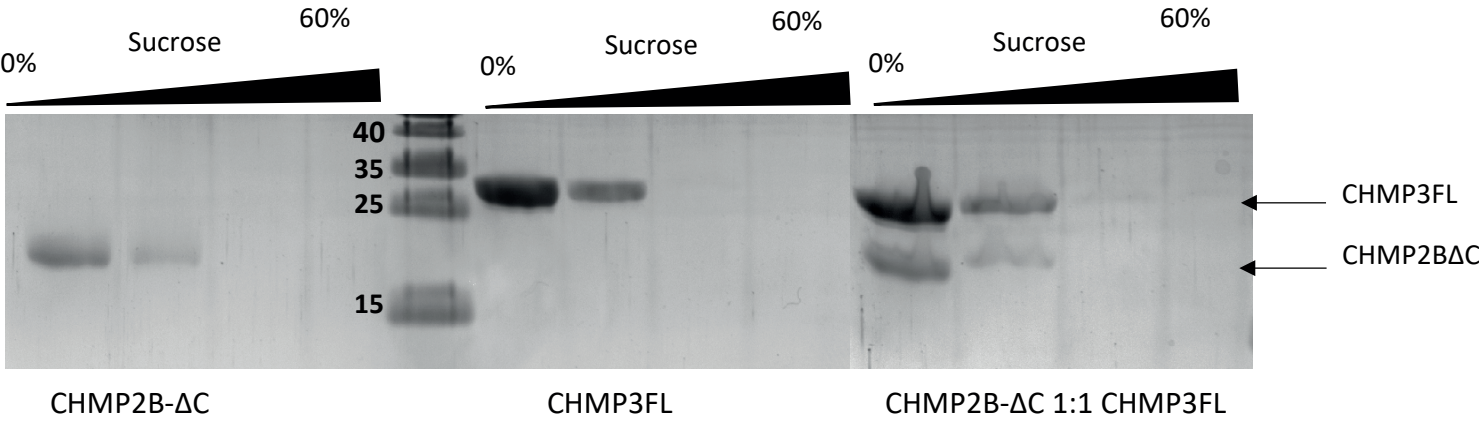

Supplement: Supplementary file 7 — Additional file 7: Figures S6. Test of mutual interaction between CHMP2B and CHMP3 in solution. CHMP2B-ΔC (first gel) and CHMP3-FL (second gel) in Hepes Buffer have been deposited on a sucrose gradient. 100 μL of CHMP2B-ΔC at 10 μM has been incubated with 100 μl CHMP3FL at 10 μM and deposit on a sucrose gradient (third gel). No aggregation is observed on the bottom of the gradient. The presence of CHMP3 does not induce CHMP2B aggregation. [file 12915_2021_983_MOESM7_ESM.pdf]
