# Supplementary material for: The ESCRT-III isoforms CHMP2A and CHMP2B display different effects on membranes upon polymerization
Source: BMC Biol. 2021 Apr 8;19:66. doi: 10.1186/s12915-021-00983-9 (PMC8033747; doi:10.1186/s12915-021-00983-9)
Supplement: Supplementary file 4 — Additional file 4: Figure S4. Deformation of bare vesicles and vesicles covered with CHMP2A + CHMP3 or CHMP2B + CHMP3. HS-AFM images of a vesicle covered with CHMP2A and CHMP 3 (left column) and a vesicle covered with CHMP2B and CHMP3 proteins (right column). The deformability of the SUVs coated with corresponding proteins upon increased applied force are shown at intermediate force increments of 27% (second panels) and at higher force increment, 81% (third panels). [file 12915_2021_983_MOESM4_ESM.pdf]

SUPPLEMENTARY 4

SUV + CHMP2A + CHMP3	SUV + CHMP2B + CHMP3

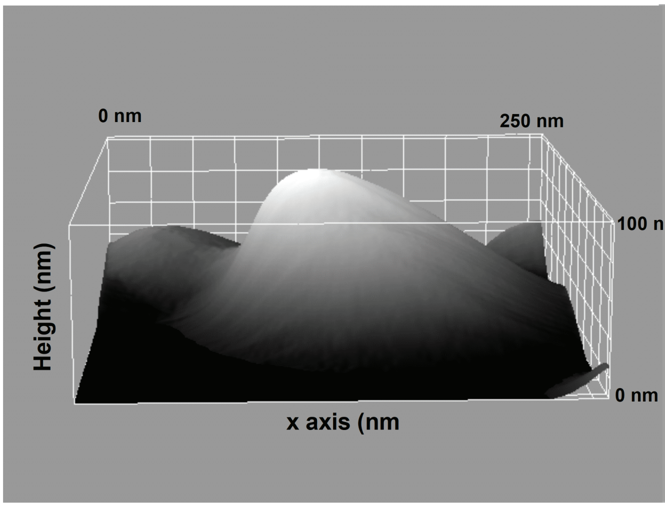

Minimum force

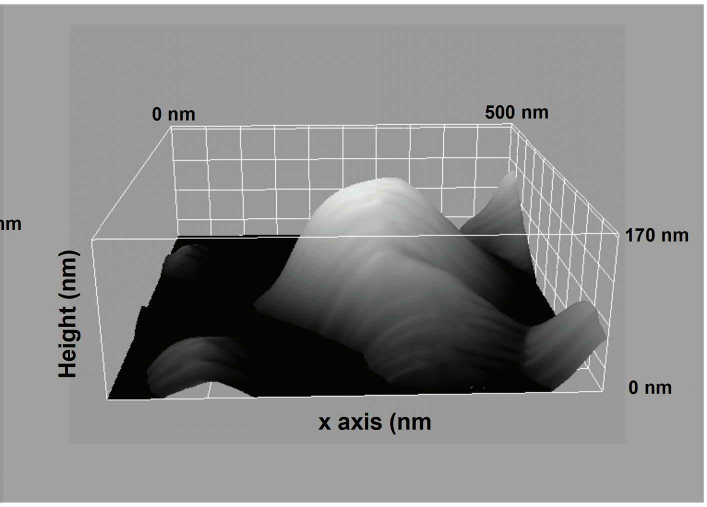

Minimum force

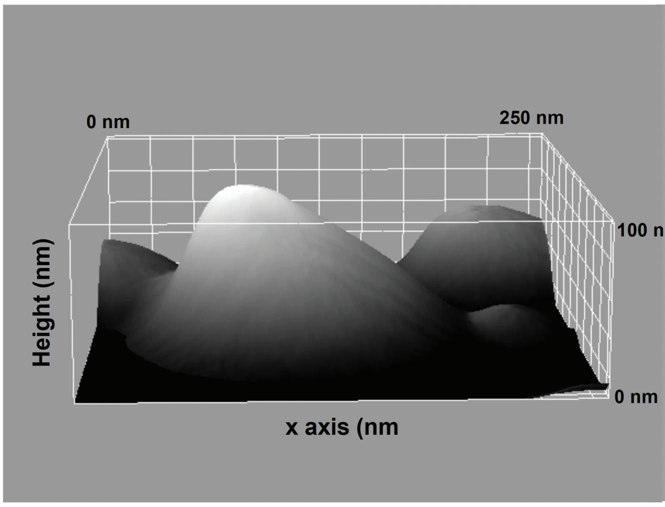

27% force increment

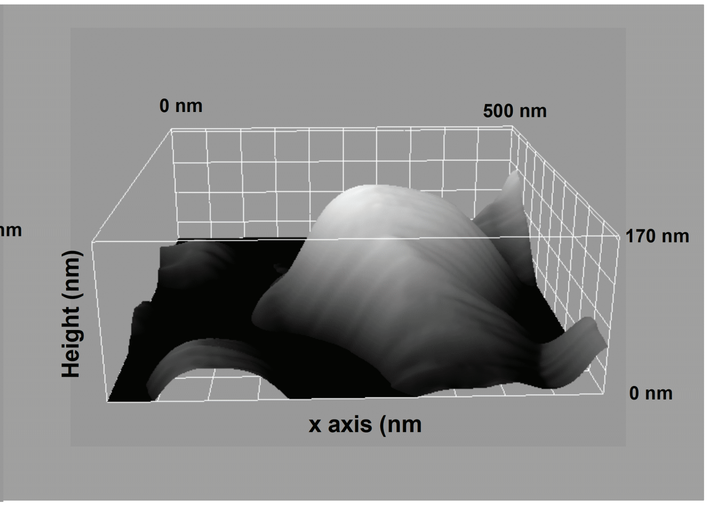

27% force increment

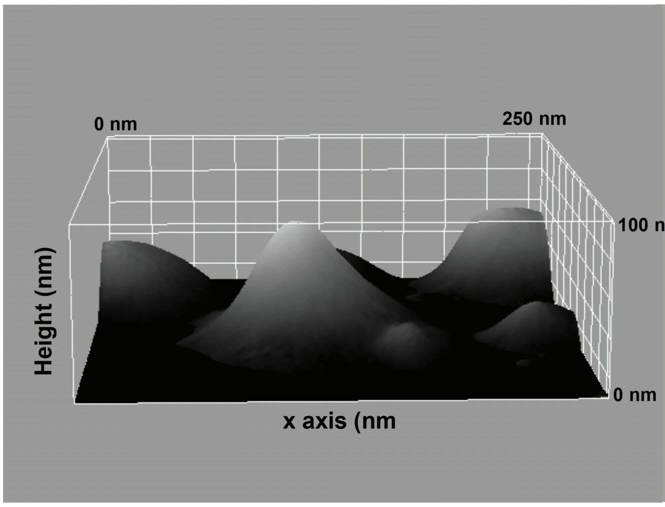

81% force increment

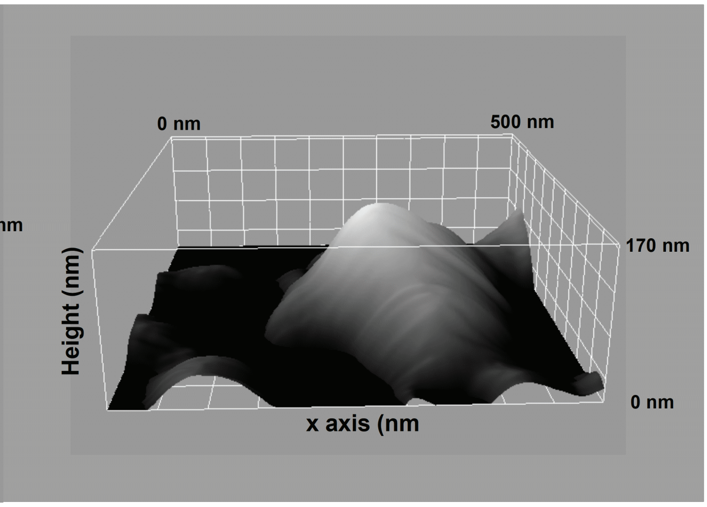

81% force increment
